# Radiation-Induced Neurodegeneration

**DOI:** 10.3390/biomedicines14020357

**Published:** 2026-02-03

**Authors:** Marialuisa Zedde, Rosario Pascarella

**Affiliations:** 1 Neurology Unit, Stroke Unit, Azienda Unità Sanitaria Locale-IRCCS di Reggio Emilia, Viale Risorgimento 80, 42123 Reggio Emilia, Italy; 2 Neuroradiology Unit, Ospedale Santa Maria della Misericordia, AULSS 5 Polesana, 45100 Rovigo, Italy

**Keywords:** neurodegeneration, radiation therapy, hippocampus, Alzheimer’s disease

## Abstract

**Background**: Radiation therapy is a critical treatment modality for craniofacial tumors and metastatic lesions, particularly gliomas. While effective, it poses significant risks of neurotoxicity, which adversely affects patient quality of life. This review aims to explore the mechanisms underlying radiation-induced neurodegeneration (RIN) and its clinical implications, focusing on the interplay between radiation exposure, cognitive decline, and potential therapeutic strategies. **Methods:** A comprehensive literature review was conducted, analyzing studies on radiation effects on the central nervous system (CNS), including mechanisms of injury, clinical outcomes, and emerging therapeutic approaches. Key areas of interest included the role of inflammation, vascular damage, neurogenesis impairment, and genetic predispositions in the context of radiation therapy. **Results:** The findings indicate that radiation induces a complex cascade of neurobiological changes, including vascular injury, microglial activation, and neurogenesis dysfunction, leading to cognitive impairments. The severity of these effects is influenced by patient age, treatment regimens, and individual genetic factors. Additionally, emerging biomarkers in cerebrospinal fluid may provide insights into individual susceptibility to radiation-induced neurotoxicity. Therapeutic strategies such as neuroprotective agents, anti-inflammatory treatments, and advanced radiation techniques show promise in mitigating cognitive decline. **Conclusions**: Radiation-induced neurodegeneration is a multifaceted process with significant implications for patients undergoing radiation therapy. The underlying mechanisms include endothelial cell apoptosis leading to blood–brain barrier breakdown, chronic inflammation, and the destruction of neural progenitor cells in the hippocampus, which collectively trigger cognitive decline and progressive degeneration. A better understanding of these mechanisms is crucial for developing effective preventative and therapeutic strategies. Future research should focus on identifying high-risk patients and exploring innovative approaches to minimize cognitive impacts while maximizing the efficacy of radiation treatment.

## 1. Introduction

Radiation therapy is essential in treating both primary tumors and metastatic lesions in the craniofacial region, particularly in cases involving gliomas [1]. Administering radiotherapy promptly after surgery can significantly improve survival rates for patients diagnosed with high-grade gliomas [2]. Common local treatment modalities for brain metastases (BM) include whole-brain radiotherapy (WBRT), stereotactic radiosurgery (SRS), and simultaneous integrated boost intensity-modulated radiotherapy (SIB-IMRT) [3,4,5]. For individuals with brain metastases originating from lung cancer, whole brain radiation therapy (WBRT) can extend median survival times to approximately three to six months, with around 10–15% of patients living beyond a year [6]. Additionally, WBRT can decrease the volume of brain metastases by roughly 60%, potentially reducing neurological deficits and enhancing overall clinical outcomes [7,8]. Despite these benefits, a major challenge remains in managing radiation-related neurotoxicity, which can significantly diminish quality of life during and after therapy [9,10]. In fact, while WBRT reduces the risk of new brain metastases, it often causes neurocognitive decline and does not consistently improve survival compared to stereotactic radiosurgery (SRS) [11]. WBRT provides superior control of brain metastases compared to SRS alone, with studies showing 6-month distant brain control rates of ~95% with WBRT vs. ~77% with SRS [12,13]. Despite better local control, randomized trials generally show no significant difference in overall survival (OS) when comparing WBRT + SRS to SRS alone [12,13]. WBRT is associated with significant risks, including cognitive impairment, memory loss, fatigue, and hair loss. Patients often experience higher rates of cognitive deterioration (e.g., 85% with WBRT vs. 52% with SRS) [12,13]. The cognitive side effects often lead to a lower quality of life, leading to a shift toward deferring WBRT in favor of SRS for oligometastatic disease. In addition, hippocampal avoidance WBRT (HA-WBRT) is used to mitigate cognitive risks [12,13,14]. In adults, long-term studies in patients treated by multiple cycles of radiotherapy (mainly metastatic brain tumors) showed that, even if the treatment itself is usually positively felt, increased levels of fatigue and significantly reduced hemoglobin and lymphocyte levels were observed [15]. In fact, the severity of radiation-induced cerebral damage is dependent on the age at the time of radiation exposure, as documented in clinical trials on childhood cancer survivors, in particular regarding neurocognitive dysfunction (intellectual disability, low school performance, IQ decline, mental disorders, psychosis) [16].

Furthermore, neurotoxic effects differ significantly between gliomas and brain metastases, with research highlighting distinct mechanisms—diffuse infiltration and altered metabolic environments in gliomas versus localized destruction from metastasis [17,18]. Current studies emphasize urgent needs to manage long-term neurocognitive, radiation, and immunotherapy-related toxicities to improve quality of life, especially as survival improves [19,20,21]. Glioma cells (e.g., glioblastoma) release factors like glutamate that cause neurotoxicity and seizures [22]. In brain metastases, research focuses on the neurotoxicity of rapid treatments like WBRT, now moving towards targeted therapies and immunotherapy (immune checkpoint inhibitors), which can cause autoimmune toxicity in the CNS. Both types of tumors are associated with blood–brain barrier (BBB) disruption, which contributes to peritumoral damage and neuroinflammation. This comparative landscape shows that while metastases often require palliative care and management of radiation toxicity, gliomas require addressing, in addition to treatments, the intrinsic neurotoxic effects of the tumor itself.

Radiation-induced damage to brain tissue can be divided into three categories according to the timing of onset and clinical features:

(1) Acute (within six weeks post-treatment);
(2) Subacute (between six weeks and six months);
(3) Late effects (months or years after therapy) [23,24,25].

Acute injuries often present with signs of increased intracranial pressure, including nausea, headaches, vomiting, and fatigue. Late effects tend to be progressive and less reversible and include conditions such as leukoencephalopathy, necrosis caused by radiation, and other neurodegenerative changes [26,27]. Neurocognitive impairment remains a prominent concern, profoundly affecting patient quality of life, and continues to be a primary focus in the management of late radiation toxicity in clinical settings.

This review aims to summarize the potential mechanisms underlying the effects of radiation therapy on neurodegeneration, along with the main clinical and neuroradiological documentation of this condition.

## 2. Mechanisms of Radiation-Induced Brain Damage

Despite the well-documented injuries associated with radiation therapy [28,29,30,31], the precise mechanisms at play remain elusive. Prior research has primarily focused on the direct harm caused to brain parenchymal cells by ionizing radiation, as well as the interactions among cells within the cerebral environment; while progress has been made, effective clinical treatments are still in development vascular leakage [32,33,34]. Recent developments have directed focus toward the immunomodulatory role of gut microbiota in neuroinflammation, especially concerning its possible effects on neurocognitive impairments [35,36,37]. Several mechanisms of gut–brain communication have been described. Mainly, radiation exposure triggers intestinal dysbiosis, characterized by an increase in pro-inflammatory bacteria (e.g., Enterobacteriaceae) and a decrease in beneficial species like Lactobacillus and Bifidobacterium. This imbalance influences neuroinflammation through three primary pathways [38,39]:

‑ Metabolic Signaling: Microbial-derived short-chain fatty acids (SCFAs) typically maintain the BBB integrity. However, recent models of radiation injury show that specific SCFA treatments may paradoxically aggravate neuroinflammation, suggesting that precise dosing or specific metabolite balances are required for neuroprotection [38,39].
‑ Immune Activation: Dysbiosis activates Toll-like receptors (TLRs) and the NF-κB signaling pathway in the gut, which can trigger systemic inflammation and the subsequent activation of microglia in the brain, leading to cognitive decline.
‑ Kynurenine Pathway (KP): Beneficial gut bacteria help regulate the KP, which balances neuroprotective vs. neurotoxic metabolites. Targeting enzymes like indoleamine 2,3-dioxygenase (IDO) is emerging as a way to reduce neurotoxic compounds post-irradiation [38,40].

While considerable progress has been made in elucidating the microbiome–gut–brain connection in various cognitive disorders, the impact of radiation-related brain injury warrants further detailed investigation and synthesis. However, therapeutic strategies are now focusing on modulating this axis, including using probiotics, prebiotics, and gut microbiota depletion to mitigate neuroinflammation [41,42].

Multiple factors influence Radiation-Induced Brain Injury (RIBI), including treatment-specific parameters, tumor features, and individual patient characteristics. A study by Colaco et al. [43] involved 180 patients with brain metastases (BM) originating from cancers such as lung, melanoma, breast, renal, and colorectal, who underwent stereotactic radiosurgery (SRS) combined with various systemic therapies like cytotoxic chemotherapy, targeted agents, or immunotherapy. The occurrence of radiation necrosis (RN) or any imaging changes related to the tumor was shown to be highest in the immunotherapy group (37.5%) than in the targeted therapy (25.0%) and in chemotherapy groups (16.9%), indicating a significant link between immunotherapy and increased RN risk [43]. Another retrospective study showed that lung cancer patients with BM receiving immune checkpoint inhibitors had an elevated risk of RN, particularly when these treatments were administered within three months of cranial radiotherapy [44]. Evidence suggests a complex interaction between RIBI and immunotherapy. A meta-analysis of 24 clinical trials demonstrated that combining hypofractionated radiotherapy with immunomodulatory agents enhanced both recurrence-free and overall survival rates compared to radiotherapy alone, without markedly increasing toxicity [45]. Furthermore, a retrospective analysis, involving 2540 patients with non-small cell lung cancer (NSCLC) and brain metastases from 11 institutions, indicated that the risk of RN and symptomatic RN was higher when V12 Gy exceeded 10 cm^3^ after single-session SRS combined with immune checkpoint inhibitors (ICIs) [46]. Importantly, concurrent immunotherapy did not significantly raise this risk, highlighting the importance of optimizing radiotherapy planning to reduce V12 Gy volume [46].

Combining radiotherapy with immunotherapy presents a unique challenge in the treatment of patients with BM. As clinicians navigate this complex landscape, caution becomes paramount, particularly when it comes to understanding the implications of radiographic parameters to minimize adverse outcomes.

Recent findings underscore the need for careful consideration. A multicenter retrospective review revealed that breast cancer patients with BM experienced a higher rate of symptomatic RN when treated concurrently with antibody-drug conjugates during cranial radiotherapy [47]. This alarming association raises concerns about the safety of concurrent therapies in this vulnerable population. Further insights emerged from another retrospective study that spanned from 2009 to 2022. This investigation focused on patients receiving SRS alongside targeted therapies, such as ALK/ROS1, EGFR, BRAF, and HER2 inhibitors. In this study, patients paused their targeted treatments for 2 to 4 days before and after radiotherapy. Remarkably, the results showed that the incidence of grade 2 or greater RN remained below 6%, and there were no notable differences linked to specific agents used [48]. This suggests that careful timing might mitigate some risks associated with concurrent therapies. In a separate analysis, Daniel et al. [49] examined 149 patients with lung adenocarcinoma BM treated with SRS. Their findings indicated a correlation between necrosis on imaging and the use of pemetrexed, prompting further discussions about the risks associated with specific chemotherapy agents. Moreover, a comprehensive review of 2843 patients provided a broader perspective on the issue. This large-scale study observed no significant difference in the 12-month cumulative incidence of RN when systemic therapy was administered concurrently with radiotherapy. This finding suggests that combined cytotoxic treatments do not substantially increase toxicity, challenging some preconceived notions about the safety of concurrent management strategies [50].

As the field continues to evolve, these insights emphasize the importance of a nuanced approach to treatment. By carefully considering the timing and combination of therapies, healthcare providers can strive to optimize outcomes while minimizing the risk of radiation necrosis in patients facing the complexities of brain metastases.

Overall, chemotherapy shows a relatively weak connection to RIBI. The HyTEC study revealed that the risk of symptomatic RN increased with the volume of tissue exposed during 12 Gy single-fraction SRS—specifically, risks rose by approximately 10%, 15%, and 20% at exposure volumes of 5 cm^3^, 10 cm^3^, and over 15 cm^3^, respectively. This emphasizes the importance of maintaining the V12 Gy volume below 10 cm^3^ [44,51,52]. Another comprehensive retrospective analysis indicated that a cumulative dose exceeding 75.7 Gy is associated with a higher likelihood of RIBI in lung cancer patients treated for brain metastases [53]. Although re-irradiation might contribute to increased risk, definitive conclusions are still pending as ongoing studies continue.

Data from a comprehensive analysis of 388 patients undergoing radiosurgery between 2004 and 2020 revealed a concerning 15.7% incidence of RN. This study highlighted a clear correlation between higher radiation doses and an increased risk of RN, with hazard ratios around 1.3 and a staggering 180% increase in risk observed when comparing doses of 14 Gy to 20 Gy [53]. In the quest to improve patient outcomes, further investigations have explored various treatment modalities. One notable study compared late grade 5 complications in patients with recurrent nasopharyngeal carcinoma. The findings indicated that hyperfractionation was associated with significantly fewer severe adverse events compared to standard fractionation schedules [54]. This suggests that treatment protocols can be optimized to reduce the risk of severe complications. Additionally, a phase III clinical trial assessed cognitive outcomes in patients with one to three brain metastases. This trial compared the effects of SRS alone versus SRS combined with WBRT. Remarkably, the SRS-only group experienced less cognitive decline at the three-month mark, despite no significant differences in overall survival [55]. These studies collectively enhance our understanding of treatment effects and inform personalized approaches for managing brain metastases in clinical practice. However, the correlation between cancer type and radiological brain injury is not consistently observed across all studies. Some research indicates that patients with renal and lung adenocarcinomas are at an elevated risk for RN. Specifically, those with lung adenocarcinoma who harbor EGFR mutations or ALK rearrangements appear particularly susceptible to RN. Similarly, in breast cancer, individuals with estrogen receptor-positive, progesterone receptor-positive, and HER2-amplified tumors exhibit a higher likelihood of developing RN [56,57,58].

Tumor size also plays a crucial role in determining RIBI. The RTOG 90-05 study assessed dose tolerance in SRS for recurrent primary brain tumors or cerebral metastases, focusing on severe neurotoxicity, classified as grade 4 to 5, or irreversible grade 3 neurotoxicity occurring within three months post-treatment. This pivotal study established dose limits based on tumor size: 24 Gy for tumors ≤ 2 cm, 18 Gy for tumors measuring 2.1–3 cm, and 15 Gy for tumors measuring 3.1–4 cm [57]. The findings reinforced the notion that larger tumors are linked to increased neurotoxicity, highlighting the need for individualized dosimetry to optimize treatment efficacy while minimizing neurotoxicity. Multivariate analyses further indicated that neurotoxicity of grades 3, 4, or 5 was associated with larger tumor diameters, with neurotoxicity being notably more likely in tumors ranging from 21 to 40 mm compared to those under 20 mm [59].

Research has increasingly highlighted that the neurotoxic effects of radiation are significantly affected by both the dose received and the age at which exposure occurs. This concern is particularly alarming for children, whose developing brains are notably sensitive to radiation. Such exposure can lead to severe long-term neurological complications, making the management of pediatric patients a critical area of focus [60,61]. Many young survivors of radiation therapy report experiencing significant neurocognitive impairments, which can manifest as motor, intellectual, visual, and psychological dysfunctions [62].

Conversely, the understanding of radiation effects in older adults is still limited, largely because they are often excluded from clinical trials. Nonetheless, advancing age has been shown to significantly increase the risk of cognitive decline following WBRT. A study conducted by Chan et al. [63] demonstrated that elderly patients are particularly vulnerable; all individuals aged 70 and older exhibited cognitive deterioration after undergoing WBRT. This stark finding underscores the importance of considering patient age when evaluating the risks and benefits of radiation therapy and highlights the urgent need for further research into potential strategies for mitigating these adverse effects. In a comprehensive investigation aiming to explore the relationship between genetic variations and disease phenotypes, researchers conducted a genome-wide association analysis. They identified a significant mutation site (rs17111237) in the promoter region of the CEP128 gene located on chromosome 14, which is associated with the development of RIBI in patients with nasopharyngeal carcinoma. Notably, this mutation correlated with increased expression of the CEP128 gene in individuals carrying high-risk alleles compared to those with low-risk alleles [64]. After accounting for other clinical risk factors, the study determined that patients with high-risk genotypes were three times more likely to develop RIBI within five years than those with protective genotypes. Furthermore, the study delved into the relationships between APOE genotypes, baseline serum protein levels, and subsequent declines in neurocognitive function induced by radiation. Contrary to initial expectations, no significant correlation was found between APOE genotypes and neurocognitive impairments at the three-month follow-up. However, a strong association emerged between lower baseline serum concentrations of ApoJ, ApoE, or ApoA proteins and exacerbated neurocognitive deficits. Elevated levels of amyloid-beta (Aβ 1–42) further supported this trend. Importantly, reduced levels of ApoJ were significantly linked to cognitive decline following WBRT [65].

These empirical findings enhance our understanding of the complex interactions among genetic predispositions, serum protein levels, and the effects of radiation therapy. They pave the way for the development of precision medicine strategies aimed at mitigating cognitive impacts during oncological interventions, emphasizing the need for personalized approaches to treatment.

RIBI is currently recognized as a multistage process that encompasses various cellular elements. Radiation therapy, while a crucial treatment for various cancers, has multifaceted effects on brain health that warrant careful consideration. One significant consequence is vascular injury, which can lead to impaired brain perfusion. This disruption of blood flow compromises the delivery of essential nutrients and oxygen to brain tissue, potentially resulting in severe neurological deficits. In addition to vascular damage, radiation therapy inflicts harm on glial cells, which play a vital role in maintaining homeostasis within the central nervous system. The damage to these support cells triggers a cascade of neuroinflammation, further exacerbating the risk of cognitive decline and other neurological complications.

Moreover, radiation exposure has been linked to the induction of cellular senescence. This process contributes to an aging phenotype, wherein cells lose their ability to divide and function properly. As a result, the brain may exhibit signs of accelerated aging, including cognitive impairments and decreased regenerative capacity. Lastly, radiation can dysregulate neural stem cell function, which is crucial for maintaining a balance between neural regeneration and repair. Disruption of these processes can hinder the brain’s ability to recover from injury and adapt to changes, ultimately impacting overall cognitive function and resilience. Together, these mechanisms highlight the complex interplay between radiation therapy and brain health, underscoring the need for targeted strategies to mitigate these adverse effects and preserve neurological function in patients undergoing treatment.

**Figure 1:**
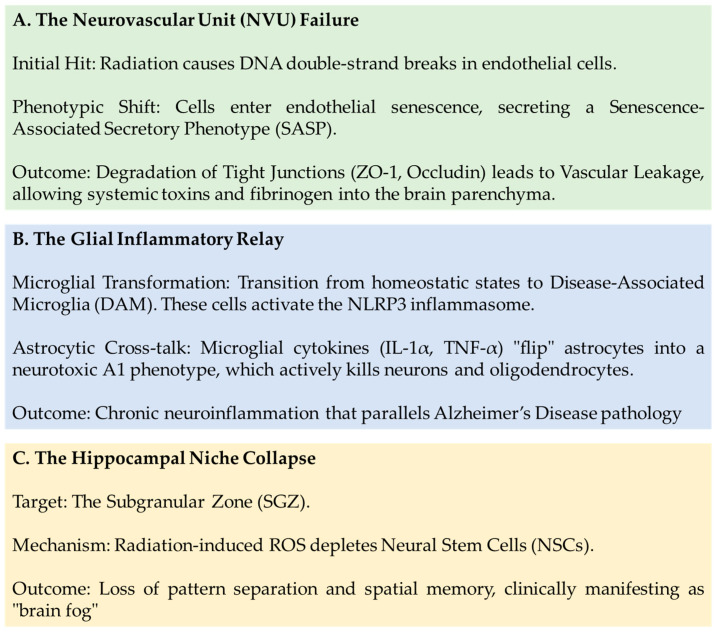
Schematic Concepts for RIBI through three primary “hubs” of toxicity.

RIBI is best understood through a Multicellular Cascading Model. The process is not a linear event but a series of interconnected feedback loops involving the vasculature, the immune system, and the neurogenic niche [66,67,68,69,70,71], as illustrated in Figure 1.

A model of RIBI cascade is provided in Table 1.

### 2.1. Vascular Damage

RIBI initiates with a biphasic pattern of vascular changes. The acute phase is typically characterized by apoptosis [72]. Following this, the chronic phase, which can last several months, is defined by capillary collapse, thickening of the basement membrane, and a halt in endothelial clonogenic activity [73,74]. The loss of endothelial cells may result in vascular leakage, which could contribute to cognitive decline [75]. Research increasingly identifies vascular leakage as the primary driver of late-delayed radiation-induced neurodegeneration. This leakage is not merely a side effect but a critical initiator that transforms acute radiation damage into chronic, irreversible brain injury [76,77]. Two distinct phases of vascular failure following irradiation have been identified:

‑ Acute Phase (<24 h): Radiation triggers immediate endothelial cell apoptosis, particularly in the highly sensitive capillary beds. This creates physical gaps in the BBB, leading to transient leakage.
‑ Late-Delayed Phase (Months to Years): Chronic leakage is driven by endothelial senescence and “sterile inflammation”. Senescent cells remain in the vasculature, secreting a Senescence-Associated Secretory Phenotype (SASP) that continuously degrades tight junction proteins like ZO-1 and VE-cadherin [78,79,80,81].

Another mechanism is the ROS-Mitochondrial-Immune Axis, identifying the role of mitochondrial DNA (mtDNA) leakage [79,82]. In particular,

‑ Radiation-induced oxidative stress damages mitochondrial membranes within endothelial cells and microglia.
‑ Escaped mtDNA activates the cGAS-STING pathway, which triggers a massive type I interferon response.
‑ This persistent inflammation prevents the repair of the BBB and forces the neurovascular unit into a state of “accelerated aging” [79,82].

Vascular leakage leads to neurodegeneration through several cascading events [79,80,83]:

‑ Vasogenic Edema: Plasma proteins extravasate into the brain parenchyma, increasing intracranial pressure and causing white matter necrosis.
‑ Neurotoxic Influx: Leakage allows systemic neurotoxic agents and inflammatory cells (M1 macrophages) to enter the brain, further activating resident microglia.
‑ HIF-1α and Aberrant Angiogenesis: Tissue hypoxia triggers HIF-1α, which stimulates the secretion of VEGF. This results in “fragile” neovascularization—newly formed vessels that are inherently leaky, creating a feedback loop of edema and ischemia.

Summarizing, irradiation induces both apoptosis and senescence in microvascular endothelial cells [83]. Advanced vascular changes, such as dilation of capillaries and microvessels, along with thickening of vessel walls, can lead to ischemic strokes, cerebral microbleeds, and small vessel occlusions, causing secondary white matter necrosis and cognitive impairments [84,85,86]. In addition to the previously mentioned effects, radiation therapy also inflicts damage on the vascular tissue surrounding tumors. This damage impedes oxygen diffusion, leading to tissue hypoxia—a condition that has significant implications for tumor behavior and patient outcomes. As oxygen levels drop, the expression of hypoxia-inducible factor (HIF-1α) increases [84]. Elevated levels of HIF-1α play a pivotal role in the response to hypoxia. They stimulate reactive astrocytes to release vascular endothelial growth factor (VEGF), a potent pro-angiogenic factor [87,88]. This process initiates abnormal neovascularization, wherein new blood vessels form in response to the hypoxic environment [89,90,91]. However, these newly formed vessels are often disorganized and fragile, characterized by high permeability. This fragility facilitates the exudation of fluid from surrounding tissues, ultimately leading to the development of cerebral edema.The presence of cerebral edema further exacerbates local tissue ischemia and hypoxia, creating a vicious cycle that can culminate in radiation-induced cerebral necrosis. This complex interplay between vascular damage, hypoxia, and abnormal angiogenesis underscores the need for vigilant management of these side effects in patients undergoing radiation therapy, as they can significantly impact both neurological function and overall treatment outcomes.

**Figure 2:**
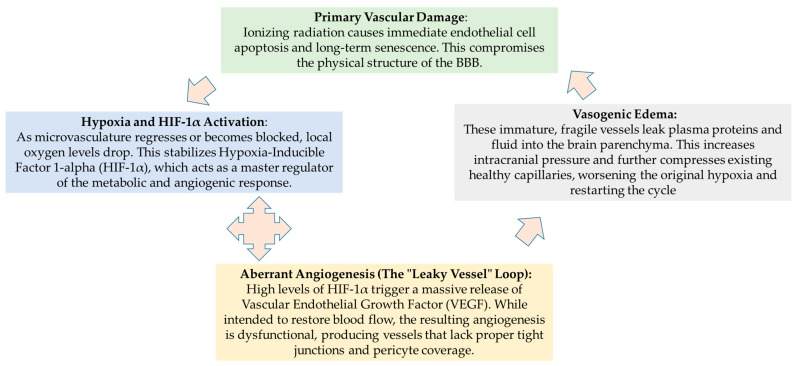
The Cyclical Pathophysiology of Radiation Injury.

The pathophysiology of RIBI is increasingly viewed as a self-sustaining cycle where vascular failure and metabolic stress reinforce one another. This cyclical process eventually leads to the chronic cerebral edema and white matter necrosis seen in late-stage neurodegeneration. The following diagrams illustrate the transition from initial endothelial damage to the chronic, self-perpetuating loops of hypoxia and aberrant angiogenesis (Figure 2).

### 2.2. Aberrant Activation or Damage of Glial Cells

Microglia, the resident immune cells of the brain, play a vital role in various functions essential for development and homeostasis. These functions include immune surveillance, regulation of inflammation, clearance and phagocytosis, neurotrophic support, and the promotion of neuroprotection and repair [92,93,94]. In their resting state, microglia remain inactive [95]. However, exposure to radiation disrupts their normal environment, prompting them to initiate a response. While this activation is crucial for addressing immediate challenges, prolonged activation can lead to chronic neuroinflammation and cognitive deficits in the later stages of RIBI.

Recent advancements in neuroimmunology have illuminated the phenotypic transformation that microglia undergo upon activation. Practically, they shift from an anti-inflammatory M2 state to a pro-inflammatory M1 state [96,97,98]. This transition is characterized by increased production of reactive oxygen species (ROS) and nitric oxide (NO), along with elevated levels of inflammatory mediators such as interleukin-1 (IL-1), tumor necrosis factor-alpha (TNF-α), interleukin-6 (IL-6), cyclooxygenase-2 (COX-2), monocyte chemoattractant protein-1 (MCP-1), and intercellular adhesion molecule 1 (ICAM-1) [99,100,101,102]. The continuous release of these pro-inflammatory factors sustains an inflammatory state within the brain microenvironment, leading to neuronal and progenitor cell death. This creates a detrimental cycle of microglial activation, inflammatory factor release, and neuronal loss [103,104,105]. Research in rodent models has shown that a single high-dose irradiation results in a sustained elevation of activated microglia and TNF-α levels, lasting for at least six months [106,107]. Moreover, after radiation-induced brain stimulation or cerebral ischemia, microglia can contribute to secondary brain injury by secreting chemokines such as CCL2 and CCL8 [108]. These chemokines attract peripheral CD8+ T cells, which then release cytotoxic agents like perforin and granzyme.

However, the understanding of microglia in radiation-induced neurodegeneration has moved beyond simple activation to a complex, multi-dimensional phenotypic transformation. Rather than a binary “on/off” switch, microglia exhibit high spatial and temporal heterogeneity, influenced by metabolic shifts and systemic immune priming [109,110,111,112]. The main molecular issues are the following ones:

1. Beyond M1/M2: Disease-Associated Microglia (DAM)

The traditional M1 (pro-inflammatory) vs. M2 (anti-inflammatory) paradigm is now considered an oversimplification. Recent single-cell RNA sequencing (scRNA-seq) has identified specific subpopulations in radiation-injured brains [109,113,114]:

‑ Persistent Priming: Radiotherapy induces “innate immune reprogramming,” making microglia more susceptible to secondary systemic challenges long after the initial exposure.
‑ Rod-Shaped Microglia: this specific morphology is a direct response to cortical hyperactivity, where they interact with neuronal dendrites to modulate synaptic inputs—a potentially neuroprotective but fragile state.
‑ DAM Profiling: In radiation-induced injury, microglia often transition into a Disease-Associated Microglia (DAM) state, characterized by the downregulation of homeostatic markers (e.g., P2ry12, Tmem119) and upregulation of phagocytic and inflammatory genes like Apoe and Trem2 [110,115].

2. Metabolic Reprogramming (The “Warburg-like” Shift) [112,116]:

‑ Glycolytic Switch: Upon irradiation, microglia shift from efficient oxidative phosphorylation to rapid aerobic glycolysis. This shift provides the quick energy needed for the production of pro-inflammatory cytokines and reactive oxygen species (ROS) but at the cost of long-term mitochondrial health.
‑ Succinate and Lactate Accumulation: Increased glycolytic flux leads to the accumulation of metabolites like succinate, which stabilizes HIF-1α, further driving the inflammatory phenotype and contributing to vascular leakage [112,117,118].

3. Chronic Neuroinflammation and Neurogenesis

Persistent activation prevents the brain’s natural repair mechanisms [119]:

‑ Inhibition of Neurogenesis: Chronically activated microglia in the hippocampus release factors that specifically inhibit the maturation of neural progenitor cells, directly linking microglial transformation to the cognitive decline seen in head and neck cancer patients after radiotherapy.
‑ Cross-talk with Peripheral Cells: Modern models show that radiation-damaged microglia recruit peripheral immune cells (monocytes/macrophages) into the brain, which “primes” the resident microglia to maintain a toxic pro-inflammatory environment even in the absence of new stimuli [102,109,120].

Astrocytes, another critical cell type in the brain, are essential for the maturation of the central nervous system (CNS). They provide crucial support to neurons, maintain homeostasis, and regulate neurotransmitter levels [121]. Typically, astrocytes are small cells with short processes. However, during pathological conditions—such as CNS injury, inflammation, or exposure to toxins—astrocytes transition from a resting state to a reactive phenotype known as astrocytic reactive hyperplasia [122]. Depending on the extent of neural tissue damage, astrocytes become activated and proliferate throughout the affected region, a phenomenon referred to as reactive astrocytosis, which can culminate in glial scarring [123,124].

Reactive astrocytes exhibit increased proliferation, cellular hypertrophy, upregulation of the intermediate filament glial fibrillary acidic protein (GFAP), and enhanced secretion of various pro-inflammatory mediators, including cyclooxygenase and a novel variant, intercellular adhesion molecule-29 (ICAM-29) [125,126]. This array of pro-inflammatory mediators facilitates various inflammatory and remodeling processes. Zhou et al. [127] demonstrated that X-ray irradiation could directly activate astrocytes in vitro, leading to reactive proliferative hypertrophy, increased GFAP expression, and heightened intracellular levels of vascular endothelial growth factor (VEGF), potentially contributing to radiation-induced brain injury.

Microglia and astrocytes do not merely activate; they engage in a coordinated temporal relay. Their roles shift from protective “first responders” in the acute phase to drivers of chronic, degenerative remodeling in the late stage. Therapeutic windows are now being defined by these stages:

1. Early Phase: Focuses on Radioprotectors to prevent initial endothelial and glial apoptosis.
2. Late Phase: Focuses on Senolytics (drugs that clear senescent cells) and A1-Inhibitors to revert astrocytes to a pro-healing (A2) state.

The following tables (Table 2 and Table 3) summarizes the differential role of microglia and astrocytes in the early and late stage of RIBI.

Oligodendrocytes, which constitute approximately 45% of the total glial cell population, play a crucial role in human white matter [128]. Numerous studies have highlighted their importance in supporting axonal metabolism [129,130] and regulating the behavior of neural networks [131,132]. A key function of oligodendrocytes is the generation and maintenance of myelin sheaths within the CNS, making them essential players in the context of RIBI. Demyelination, a hallmark of delayed radiation injury, is significantly influenced by the health and function of oligodendrocytes. Compared to other glial cells, such as microglia, oligodendrocytes exhibit greater sensitivity to radiation exposure [133,134]. Ionizing radiation has been shown to directly trigger apoptosis in oligodendrocytes, leading to cellular loss and impaired myelin production [135]. Furthermore, radiation-induced oxidative stress adversely affects oligodendrocyte maturation [136] and is linked to the onset of demyelinating neuroinflammation [137]. Oligodendrocyte precursor cells (OPCs) are also particularly vulnerable to the effects of ionizing radiation. This vulnerability may lead to a diminished capacity for these cells to differentiate into astrocytes and neurons [138]. As a result, radiation-induced damage to OPCs can indirectly compromise the functionality of other cell types within the CNS [139].

The detrimental effects on oligodendrocytes and the subsequent demyelination they cause are significant contributors to the development of delayed RN [140]. Understanding the critical role of oligodendrocytes in the context of radiation exposure underscores the importance of developing strategies to protect these cells and preserve myelin integrity, ultimately aiming to mitigate the long-term consequences of radiation therapy on brain health.

### 2.3. Loss of Hippocampal Neurogenesis

The hippocampus is critically important for learning and memory, with adult neurogenesis primarily occurring in the dentate gyrus and the subgranular zone (SGZ) of the hippocampus, as well as in the subventricular zone (SVZ) of the lateral ventricles. Research has consistently demonstrated that radiation negatively affects neurogenesis in these regions, inhibiting the differentiation of neural precursor cells (NPCs) into mature neurons, particularly in animal models [141,142]. For instance, one study found that mice subjected to 10 Gy of intracranial radiation exhibited reduced neurogenesis, which was associated with poorer cognitive performance on maze tests [53]. Further supporting the connection between NPC loss and cognitive dysfunction after radiation, studies have suggested that cognitive function can be partially restored through neural stem cell transplantation, which replaces the lost hippocampal NPCs following whole-brain irradiation in mice [143].

Numerous investigations are underway to elucidate the mechanisms by which radiation depletes NPCs in the hippocampus. One prominent hypothesis suggests that radiation triggers inflammation and microvascular damage in the SGZ and SVZ, thereby altering the microenvironment of progenitor cells in a way that inhibits their differentiation into neurons. Additionally, dysregulated signaling within hippocampal neurons—such as the downregulation of hippocampal glutamate receptor 1 and protein kinase C-gamma via Homer1a—has been shown to impair long-term potentiation, working memory, and synaptic plasticity [144]. These alterations in neuronal signaling may also lead NPCs in the hippocampus to favor glial differentiation over neuronal differentiation [145,146,147,148]. Several clinical trials indicate that the findings from animal studies may be applicable to humans. In a prospective observational study, Gondi et al. [149] enrolled adults with benign or low-grade brain tumors who received fractionated stereotactic radiotherapy. The study correlated hippocampal dose-volume histogram data with cognitive impairment, concluding that bilateral doses to the hippocampus exceeding 7.3 Gy are linked to long-term cognitive deficits. This finding supports the implementation of hippocampal avoidance strategies in radiation therapy.

In a subsequent phase II trial (RTOG 0933), Gondi et al. [150] included patients with brain metastases treated using intensity-modulated radiotherapy (IMRT), which allowed for hippocampal avoidance. Cognitive function was assessed using the Hopkins Verbal Learning Test prior to treatment and at two-month intervals for up to six months afterward. The cognitive outcomes of patients receiving hippocampal avoidance radiotherapy were compared to historical controls of patients who underwent whole-brain radiation without hippocampal avoidance. The historical control group exhibited a 30% mean relative decline in cognitive function from baseline over four months, whereas those receiving hippocampal avoidance experienced only a 7% mean relative decline. These results underscore the preservation of cognitive function when employing hippocampal avoidance strategies during radiation therapy.

### 2.4. Radiation-Induced Aging

Radiation therapy has been linked to the induction of cellular senescence, a phenomenon characterized by a permanent halt in the cell cycle, which plays a fundamental role in the aging of tissues and the progression of degenerative diseases [151]. This state of senescence is marked by an increased expression of senescence-associated β-galactosidase (SA-β-Gal) and the upregulation of specific senescence-related genes, including p21, p39, and Bcl2. Notably, these changes are particularly evident in macrophages that originate from bone marrow following exposure to radiation [152].

In vitro studies have provided compelling evidence that exposure to radiation can trigger a stress-induced premature senescence phenotype in endothelial cells. This response leads to the expression of the P16 protein, ultimately resulting in an irreversible arrest of the cell cycle. Furthermore, damage inflicted on the cerebral microvasculature by gamma irradiation accelerates the aging process in otherwise healthy tissues, which is a significant contributing factor to cognitive decline observed in nearly 50% of patients undergoing radiotherapy for tumors [153]. Additionally, research has shown that irradiated microglia increase the expression of key senescence markers, including SA-β-Gal and p16INK4a. These alterations may persist for up to a month after radiation exposure and are likely linked to the oxidative stress induced by radiation, as well as to processes such as autophagy, telomere shortening, and mitochondrial dysfunction [154].

While in vitro studies involving primary cell cultures indicate that irradiated astrocytes do not transition to a fully reactive state [155], signs of the aging phenotype remain detectable. A significant study highlighted a notable increase in astrocyte senescence within irradiated human brain tissues [156]. The implications of cellular senescence extend beyond individual cells and are reflected in tissue-level aging. In a cohort study involving 4220 individuals, which included 4148 healthy participants and 72 patients who received an average radiation dose of 30 Gy, those exposed to radiation exhibited structural changes in the brain that indicated accelerated aging compared to their healthy counterparts. Notably, subset analyses revealed that the hippocampus experienced an accelerated aging rate of 8.88 times following conventional WBRT compared to treatment protocols that spared the hippocampus [157].

Furthermore, a long-term follow-up study conducted by Remes et al. [156] tracked childhood brain tumor survivors who underwent radiation therapy over a span of 20 years. The findings indicated that their rates of ischemic infarction, microbleeds, and lacunar infarctions were comparable to or even exceeded those of the general population over the age of 70. This reinforces the concept that radiation exposure accelerates cerebrovascular aging. The role of senescence in the delayed onset of RIBI underscores the long-term consequences of radiation exposure, particularly in younger patients. This highlights the importance of identifying potential therapeutic targets to mitigate these adverse effects and improve outcomes for those affected.

### 2.5. Neurogenesis Dysfunction

Radiation-induced neurocognitive damage is notably characterized by the activation of microglia within the dentate gyrus, a critical region of the hippocampus. Concurrently, there is a marked apoptosis of myeloproliferative cells located in the SGZ of this same area, which is essential for the processes of lifelong learning and memory due to its inherent neurogenic capacity [158,159]. Neural stem cells, which are indispensable for the ongoing process of neurogenesis, face significant negative impacts from exposure to ionizing radiation. Such exposure disrupts their ability to proliferate effectively, disturbs the maintenance of their cell cycle, and ultimately undermines their overall stemness, which is crucial for their functionality [160]. Furthermore, radiation exposure interferes with the differentiation pathways of these neural stem cells, leading to alterations in the expression of various proteins that are fundamental to the generation of new neurons. Because of this exposure, a persistent decline in neurogenesis within the SGZ of the hippocampus is commonly observed [161,162,163,164,165,166].

These disruptions in neurogenesis can culminate in chronic and irreversible cognitive deficits, significantly increasing the risk of developing dementia over time. While some immediate cognitive effects may manifest shortly after radiation exposure, it is often the delayed consequences that pose a greater threat, frequently resulting in long-term cognitive impairments and the potential onset of dementia. This underscores the critical need to understand the underlying mechanisms that contribute to radiation-induced neurocognitive damage. Moreover, it highlights the importance of developing effective strategies aimed at mitigating these long-term adverse effects, particularly for individuals who undergo radiation therapy, ensuring that their cognitive health is preserved as much as possible.

## 3. Radiation-Induced Neurodegeneration

Ionizing radiation (IR) comprises subatomic particles and electromagnetic waves (photons) capable of generating charged particles, such as alpha and beta particles, gamma rays, and X-rays. While the biological impacts of IR on humans have been documented for over a decade, there is a growing interest in its effects on the CNS in clinical contexts, particularly in light of the current global threat of radiation exposure from warfare [167]. It is essential to re-evaluate the neurobiological effects of both high and low doses of IR, especially regarding the hippocampus, a radiosensitive area that houses proliferating progenitor cells [168,169].

High doses of IR can induce apoptosis and dysfunction in differentiating cells within the hippocampus, leading to changes in synaptic protein levels, dendritic complexity, and spine density [170]. Regulatory guidelines suggest that a safe acute exposure dose is below 100 mSv, or 0.1 Gy [171]. Research shows that IR inhibits neurogenesis in a dose-dependent manner (ranging from >2 Gy to 45 Gy), affecting neural stem and progenitor cells in the subgranular zone of the dentate gyrus, which may result in neuroinflammation [172,173,174]. Exposure to low doses of IR has been found to increase reactive oxygen and nitrogen species, potentially disrupting the redox balance within the CNS [175].

Acute IR exposure has a wide range of effects on brain and cognitive functions [176], including direct impacts on the nervous system and indirect damage to other systems due to CNS reactivity. Both low and high doses can induce oxidative stress, mitochondrial dysfunction, and protein degradation, leading to cellular senescence or apoptosis, which may contribute to neurodegenerative diseases.

IR triggers inflammatory responses in the brain through the activation of microglia and endothelial cells. This microglial activation results from IR-induced double-strand breaks, which activate the NFkB pathway and promote the production of inflammation-related proteins [26]. Damaged neurons release high-mobility group protein 1 (HMGB1), which interacts with TLR4 on activated microglia. Furthermore, calreticulin expressed on damaged neurons encourages phagocytosis by activated microglia. This activation also enhances the secretion of chemokines, facilitating the infiltration of peripheral macrophages across the BBB and stimulating immune responses [26].

Pro-inflammatory cytokines secreted by activated microglial cells inhibit neurogenesis in the hippocampus, disrupting neurogenic signaling pathways. Acute radiation sickness in mice has been shown to impair hippocampal functions, including learning and memory, due to inhibited neurogenesis [143]. The decrease in neurogenesis associated with IR-induced cognitive impairments is linked to a reduction in proliferating Ki-67-positive cells and doublecortin-positive immature neurons in the SGZ of the dentate gyrus.

Cells respond to stress through various pathways, including the unfolded protein response, oxidative stress response, and DNA damage response, which help maintain cellular homeostasis or trigger apoptosis [177]. Radiation exposure can cause protein misfolding and aggregation, activating stress response pathways and potentially accelerating the rates of misfolding in proteins [178,179]. This aggregation contributes to the development of neurodegenerative diseases.

In everyday life, we are exposed to IR from both natural and artificial sources, and this exposure is known to play a role in the etiology of neurodegenerative diseases (see Table 4). This review discusses the involvement of IR exposure in various neurodegenerative diseases [180].

IR can inflict varying degrees of damage to human DNA, particularly by inducing double-stranded breaks (DSBs). These DSBs hinder DNA replication in proliferating cells, causing a cell cycle arrest during the S-phase of development. If these breaks are not repaired, they can ultimately lead to cell death. Additionally, exposure to IR generates reactive oxygen species (ROS), which contribute to oxidative stress and are indirectly associated with DNA damage.

Cells possess intricate repair mechanisms that respond to DNA damage by pausing the cell cycle at specific checkpoints. This pause allows time for repair processes to occur, thereby preventing the cell from progressing through the cycle until any damage is resolved. Key signaling molecules, notably ataxia telangiectasia mutated (ATM) and RAD3-related (ATR) kinases, are activated in response to DNA damage and play crucial roles in orchestrating the DNA damage response signaling pathway. These kinases are responsible for regulating numerous downstream processes, including the phosphorylation of the histone variant H2AX (referred to as γH2AX), which is essential for recruiting DNA repair proteins to sites of damage and activating checkpoint proteins that halt cell cycle progression.

However, the presence of ROS can significantly disrupt the cell cycle, diminish cell viability, and cause oxidation of proteins and lipids. This oxidative stress ultimately leads to increased cell death. For example, research conducted by Koturbash et al. [200] demonstrated that exposure to low doses of IR results in DNA damage, as evidenced by a heightened incidence of DSBs along with associated behavioral changes in affected organisms. Moreover, it has been shown that low-dose radiation can alter the expression of genes that play critical roles in cell cycle regulation and DNA synthesis and repair processes [201].

Brain cells, which are typically non-proliferative, may be particularly vulnerable to the accumulation of unrepaired DNA lesions. Research indicates that exposure to low doses of radiation (less than 50 cGy) is associated with neurocognitive deficits, including impairments in learning and noticeable behavioral changes. Patients undergoing radiation therapy for cancer frequently report experiencing chronic fatigue and depression as side effects of their treatment. Particularly concerning are the significant neurocognitive effects observed in children exposed to radiation, which often manifest as declines in academic performance and reductions in intelligence quotient (IQ). Recent studies have increasingly focused on the intricate relationship between DNA damage and the mechanisms of repair in the context of neurological diseases. Numerous neurodegenerative conditions have been linked to deficiencies in repairing both single-strand breaks and double-strand breaks in DNA. In diseases such as Parkinson’s disease (PD) and Alzheimer’s disease (AD), defects in the DNA repair processes can lead to the abnormal accumulation of DNA damage within neurons. This accumulation ultimately results in premature neuronal death, highlighting the critical importance of effective DNA repair mechanisms in maintaining neuronal health and function [201,202].

To comprehend the role of DNA damage in the pathology of neurodegenerative diseases, it is essential to identify specific lesions that accumulate in affected neurons and to elucidate the molecular mechanisms that impede their repair.

### 3.1. Radiation Induced Cognitive Decline

RT is a common treatment for cancers of the CNS, but it can negatively affect cognitive function, leading to radiation-induced cognitive decline (RICD). RICD is characterized by impairments in memory, attention, and other cognitive abilities, primarily resulting from damage to brain white matter, inflammation, and oxidative stress. The multifactorial nature of RICD complicates its understanding and management, with various mechanisms of injury including neurogenesis impairment, oxidative stress, neuroinflammation, and alterations in dendritic structure and vascular integrity. Despite these challenges, potential solutions such as neuroprotective agents, cognitive rehabilitation, advanced imaging techniques, proton therapy, and personalized medicine are being explored. Emerging therapeutic strategies, including stem cell therapy and regenerative medicine, show promise for repairing or replacing damaged brain tissue. Addressing RICD is crucial for improving the quality of life among cancer survivors [203].

RT is widely utilized for treating CNS malignancies, but it is associated with cognitive decline, termed RICD. This decline can manifest as difficulties in memory and cognitive function due to various mechanisms that remain poorly defined. RICD is recognized as a late effect of RT, affecting a significant number of patients—30% within four months post-treatment and potentially 50% after six months. Maintaining cognitive function is vital for patients, as cognitive decline often correlates with decreased functional independence [204].

The mechanisms of injury are:

1. Neurogenesis:
  ○ Neurogenesis, the process of generating new neurons, is crucial for maintaining brain function and occurs primarily in the hippocampus and lateral ventricles. Ionizing radiation has been shown to reduce neurogenesis, particularly in the hippocampus. This reduction is thought to result from radiation directing neural progenitors to differentiate into astrocytes instead of neurons, making it difficult for the brain to replace damaged neurons [107,205].
2. Oxidative Stress and Neuroinflammation:
  ○ RT induces oxidative stress characterized by an increased production of ROS, which can overwhelm cellular repair mechanisms. Antioxidants, such as nigella sativa oil and thymoquinone, have been shown to mitigate this oxidative damage following radiation exposure [206].
  ○ The radiation-induced increase in free radicals can activate inflammatory pathways, leading to neuroinflammation, particularly in the hippocampus, where microglial activation occurs. This inflammation creates a feedback loop that can inhibit neurogenesis and exacerbate cognitive decline [170].
3. Dendritic Structure Alterations:
  ○ Dendrites are critical for synaptic function, and alterations in their structure can significantly affect cognitive abilities. Radiation exposure can lead to changes in dendritic spine density and morphology, affecting synaptic communication. Studies have shown that radiation increases dendritic spine density, which may lead to excitotoxicity through enhanced glutamate signaling [207].
4. Vascular Effects:
  ○ RT can cause vascular damage, including endothelial cell death and thrombus formation, leading to complications such as microangiopathy and ischemia. These vascular changes can result in excitotoxicity due to increased extracellular glutamate levels, further compromising cognitive function [208].

On the other hand, the proposed mitigation strategies are:

1. Stem Cell Therapy:
  ○ Stem cell therapy is being explored as a way to counteract radiation-induced neurogenesis damage. Studies indicate that transplanting stem cell-derived oligodendrocyte progenitors can improve cognitive function and promote neurogenesis in irradiated animals [207,209].
2. Anti-Inflammatory Agents:
  ○ Anti-inflammatory agents, particularly nonsteroidal anti-inflammatory drugs (NSAIDs) like indomethacin, have been shown to reduce microglial activation and improve cognitive function in irradiated models by normalizing endothelial inflammation [210,211].
3. Memantine:
  ○ As a noncompetitive NMDA receptor antagonist, memantine is used to prevent RICD by inhibiting excessive glutamate binding, thus reducing excitotoxicity. Clinical studies have shown that memantine can improve cognitive function in patients receiving whole-brain radiation therapy [212,213,214,215,216].
4. Cyclooxygenase-2 (COX-2) Inhibitors and Erythropoietin (EPO):
  ○ COX-2 inhibitors like celecoxib help maintain blood–brain barrier integrity, reducing brain injury from radiation. EPO has neuroprotective effects in various neurological disorders, showing promise in mitigating cognitive decline post-radiation [215,216,217].
5. Cognitive Training and Memory Strategies:
  ○ Cognitive interventions and memory strategies have shown efficacy in improving cognitive function in cancer survivors. Programs focusing on executive function and memory can provide significant benefits, particularly in younger patients [217].
6. Proton Therapy:
  ○ Proton therapy offers a precise radiation treatment option that minimizes damage to surrounding healthy tissue. Studies have suggested that it can prevent cognitive decline associated with traditional radiation therapy [217].

Understanding RICD requires considering patient-related factors (age, baseline cognitive status), disease-related factors (tumor location, overall disease burden), and treatment-related factors (surgical interventions, chemotherapy, and specifics of radiation therapy). Identifying high-risk patients through neurocognitive tests and biomarkers is crucial for improving outcomes.

Improving cognitive function after radiation therapy involves identifying high-risk patients. Biomarkers such as white matter hyperintensities can indicate RICD risk, and advanced imaging techniques like diffusion tensor imaging (DTI) can assess brain changes pre- and post-treatment. Studies have shown correlations between specific imaging changes and cognitive decline, emphasizing the need for early identification and intervention strategies.

The mechanisms underlying RICD include neurogenesis impairment, oxidative stress, neuroinflammation, dendritic structure alterations, and vascular changes. These entire mechanisms impact cognitive decline, through a synergistic, multi-cellular cascade where vascular damage and glial dysfunction act together, rather than in isolation, to accelerate cognitive decline. In fact, radiation damages endothelial cells and disrupts the BBB, leading to ischemia and inflammation, which concurrently activates astrocytes and microglia. These activated cells release pro-inflammatory cytokines that further impair neurogenesis, damage oligodendrocytes, and perpetuate chronic neuroinflammation [80,218,219]. The main synergistic mechanisms resulting in cognitive decline are:

1. Vascular-Glial Crosstalk: Damage to the microvasculature (endothelial cells) causes BBB breakdown, allowing peripheral immune cells to infiltrate the brain. This infiltration, combined with the release of cytokines (e.g., IL-1beta, TNF-alpha) from activated microglia, promotes chronic inflammation.
2. Neuroinflammation & Structural Damage: Activated microglia/astrocytes (gliosis) and vascular dysfunction work together to inhibit neurogenesis in the hippocampus and destroy white matter. This reduces the brain’s capacity for repair, leading to diminished memory and executive function [220].
3. Chronic Oxidative Stress: Both vascular injury and glial dysfunction enhance the production of ROS, causing a sustained, damaging, and inflammatory microenvironment.

This interplay means that even without significant gross structural damage, early vascular and glial changes can cause functional decline, often progressing to permanent cognitive impairment, particularly in the hippocampus, due to the interconnectedness of these pathological pathways [32].

Although these mechanisms highlight the detrimental effects of radiation therapy, they also present opportunities for intervention through neuroprotective agents, cognitive training, and advanced radiation therapy techniques. Future research should focus on exploring these strategies to mitigate cognitive decline in patients undergoing radiation therapy.

Another issue is prophylactic cranial irradiation (PCI). PCI is used in cancer treatment but is associated with potential neurotoxic effects on the brain. A study investigates whether CSF biomarkers can characterize the neurochemical response to PCI, aiming to identify individual susceptibility to radiation-induced neurotoxicity [221]. A prospective clinical study was conducted on patients with small cell lung cancer (SCLC) undergoing PCI. Participants were assessed before treatment and again at 3 and 12 months post-irradiation. CSF biomarkers for neuroaxonal damage, neuroglial activation, and amyloid beta (Aβ)-related processes were analyzed. Patients underwent MRI and Mini-Mental State Examination (MMSE) to evaluate cognitive function. Eighteen patients were initially included, but only 11 completed the study. Thirteen age- and sex-matched controls were also analyzed. The following results were found:

▪ Neurofilament Light (NFL) and T-tau: Elevated levels were observed after PCI, indicating neuronal injury. NFL increased by 120% and T-tau by 50% in patients without metastases at 3 months post-PCI.
▪ Amyloid Precursor Proteins: Levels of secreted amyloid precursor proteins (sAPPa and sAPPb) decreased significantly (44% and 46%, respectively) after PCI and continued to decline for a year.
▪ Neuroglial Markers: YKL-40 and glial fibrillary acidic protein (GFAP) levels increased significantly after treatment, suggesting neuroglial activation.
▪ Cognitive Function: Despite detectable neurochemical changes, the MMSE did not indicate cognitive decline, suggesting that more sensitive cognitive assessments are needed.

The study found that PCI induces significant neurochemical changes indicative of neuronal and neuroglial injury, even at moderate radiation doses (20–30 Gy). The results highlight the need for biomarkers that could predict individual susceptibility to long-term neurotoxic effects. While the study did not find cognitive decline via MMSE, it emphasizes the necessity for more sensitive neurocognitive evaluations in future research [221].

The findings suggest that CSF biomarkers can reflect neurotoxic effects following PCI. Future research should involve larger patient cohorts and more detailed neurocognitive assessments to further explore these neurochemical changes and their implications for cognitive function.

A recent systematic review investigated the factors affective neurocognitive decline in patients with lung cancer who underwent prophylactic irradiation [222]. PCI is a treatment that reduces the incidence of brain metastases in lung cancer patients but poses a risk for neurocognitive decline. This systematic review aims to identify risk factors associated with cognitive impairment following PCI. Out of 203 records screened, 20 studies met inclusion criteria, encompassing 3553 patients (858 with non-small cell lung cancer (NSCLC) and 2695 with small cell lung cancer (SCLC)). About 73.6% of these patients received PCI. The incidence of mild/moderate cognitive decline after PCI ranged from 8% to 89%, while those without PCI reported a decline of 3.4% to 42%. Notably, 23% to 95% of patients had cognitive impairment at baseline. The following risk factors for neurocognitive decline were identified:

▪ Age: Older age (>60 years) was consistently linked to higher cognitive decline risk.
▪ PCI Dose: Higher doses (e.g., 36 Gy) correlated with increased cognitive impairment.
▪ Treatment Regimen: Twice-daily PCI was associated with greater cognitive decline.
▪ Timing of Assessment: Neurocognitive function assessments varied widely, leading to inconsistent data quality.

The findings suggest that age, PCI dose, treatment regimen, and timing could influence cognitive outcomes after PCI in lung cancer patients. However, the review highlighted the lack of rigorous data, as most trials did not prioritize cognitive assessment, leading to high bias risk. Compliance in cognitive testing decreased significantly over time, further complicating the reliability of results [222].

Recent research into radiation-induced neurodegeneration has definitively linked impaired hippocampal neurogenesis to the specific “brain fog” and memory deficits observed in both animal models and human cancer survivors. As previously pointed out, the transition from acute neuroinflammation to chronic cognitive dysfunction is mediated by the destruction of the SGZ niche through the following issues:

1. The Hippocampal Neurogenic Niche Collapse

Radiation exposure is uniquely toxic to Neural Stem Cells (NSCs) and Progenitor Cells in the SGZ of the dentate gyrus, producing:

‑ Acute Depletion: Within 24–48 h of irradiation, there is a massive wave of apoptosis among proliferating neuroblasts.
‑ Microenvironmental Shift: Long-term, the niche transforms from a pro-neurogenic environment to an inflammatory one. Activated microglia release IL-6 and TNF-α, which actively suppress the differentiation of remaining stem cells into functional neurons, forcing them instead toward a gliogenic (astrocyte-forming) fate [211].

2. Behavioral Manifestations: From Models to Humans

The loss of these new neurons manifests in specific cognitive domains that mirror the clinical symptoms of “chemobrain” or “rad-brain” (Table 5).

3. Molecular Link: The VEGF-BDNF Deficit

A major breakthrough is the identification of the Vascular-Neurogenic Coupling failure.

‑ Because radiation causes vascular leakage (as discussed previously), the delivery of systemic BDNF (Brain-Derived Neurotrophic Factor) and VEGF to the hippocampus is disrupted.
‑ Without these trophic supports, new neurons fail to integrate into existing circuits (synaptogenesis), leading to the “shrunken” hippocampal volume frequently seen on high-resolution 7T MRI in post-radiation patients [225].

4. Therapeutic Interventions

‑ Physical Exercise & Environmental Enrichment: Current protocols emphasize aerobic exercise, which is proven to upregulate endogenous BDNF, partially restoring neurogenesis even after moderate radiation doses [225].
‑ Pharmacological Rescuers: Trials are currently evaluating PPARδ agonists to mitigate the microglial inflammation that halts neurogenesis, aiming to preserve spatial memory during cranial radiotherapy.

### 3.2. Role of Ionizing Radiation in Alzheimer’s Disease

Given the predicted doubling of AD prevalence over the next two decades, it is crucial to understand the molecular pathogenesis of the disease and explore contributing factors that may aid in prevention strategies. Numerous studies have documented the effects of IR on brain health, suggesting that exposure to IR may facilitate the progression of AD.

The amyloid precursor protein (APP) plays a crucial role in the pathogenesis of AD by generating 4.5 kDa peptides known as amyloid-beta (Aβ) proteins [226]. An imbalance between the production and clearance of Aβ leads to its abnormal accumulation in the brain. This accumulation is closely associated with oxidative stress, the formation of neurofibrillary tangles (NFTs), and subsequent neuronal loss [227]. Such a cascade of pathological events ultimately contributes to the cognitive impairments that characterize AD [228]. While neurons exhibit a certain degree of resilience against the cytotoxic effects of radiation, numerous studies have shown that even lower doses of IR can induce cognitive dysfunction without causing significant observable morphological changes. In contrast, exposure to higher doses of radiation tends to result in more pronounced microscopic alterations within neural tissues [229,230].

Research conducted by Cherry et al. [231] specifically investigated the effects of iron particle irradiation on a mouse model of AD (APP/PS1). After a six-month period of exposure to iron radiation at doses of 1 GeV/m (10 and 100 cGy), the APP/PS1 mice exhibited notable reductions in cognitive abilities. These impairments were assessed through novel object recognition tests and contextual fear conditioning, both of which are commonly used to evaluate memory and learning in animal models. Furthermore, an increase in Aβ plaque pathology was observed in the male mice subjected to irradiation. Immunohistochemical analysis further revealed signs of endothelial activation following exposure to the 100 cGy dose. This finding suggests potential alterations in the trafficking of Aβ across the blood–brain barrier, which may play a significant role in the observed increase in plaque pathology.

Belka et al. [232] proposed that IR exposure leads to elevated expression levels of pro-inflammatory markers such as interferon-gamma (IFN-γ) and tumor necrosis factor-alpha (TNF-α), as well as adhesion molecules like ICAM-1 and E-selectin [233]. Lowe et al. [234] demonstrated that low doses of IR can trigger gene modulation distinct from that of higher doses, impacting brain functions related to memory, learning, and cognition. Their findings indicate that global gene variations in the brains of irradiated mice resemble those observed in AD patients.

The long-term consequences of IR exposure include brain atrophy and neurological decline, which have been observed in patients undergoing radiation therapy, even in the absence of recurrent or residual brain tumors. Some reports have suggested that dementia can be detected in a subset of long-term survivors of brain tumors treated with radiotherapy [235]. MRI and CT imaging have revealed widespread damage to cerebral white matter and progressive brain atrophy [236]. Neurological deficits resulting from high-dose radiation are thought to arise from neural loss and demyelination, leading to cognitive and neurological impairments. These cognitive defects following IR exposure are often associated with impaired neurogenesis.

The effects of radiation on the CNS are particularly pronounced in children compared to adults, with IR-induced cognitive effects manifesting as more severe learning disabilities in younger populations. Studies of survivors of the atomic bombings in Hiroshima and Nagasaki indicate that exposure during critical developmental periods can have detrimental effects on brain development. Data suggest significant cognitive impairments, including severe intellectual disability and variations in IQ and academic performance, especially in those exposed during specific gestational weeks [237].

Research by Rola et al. [142] involved irradiating the brains of 21-day-old C57BL/J6 male mice with doses ranging from 2 to 10 Gy to assess acute radiosensitivity in the dentate subgranular zone. Histopathological analyses revealed a dose-dependent reduction in immature neurons. In a long-term study where mice received a single dose of 5 Gy of whole-brain irradiation, significant reductions in new neuron production were observed after one and three months, while glial cells showed no changes. Notably, deficits in spatial memory retention were recorded three months after irradiation, indicating that early irradiation can lead to long-term impairments in neurogenesis associated with hippocampal-dependent memories [142].

Further investigations have shown that irradiating 10-day-old mice with 8 Gy resulted in diminished hippocampal neurogenesis and increased susceptibility of the adult brain to hypoxia-ischemia [238]. Exposure to IR in immature brains led to long-lasting alterations, such as larger infarcts and increased hemispheric tissue loss, along with heightened inflammation compared to non-irradiated brains. Other detrimental effects of IR on the brain include severe disruption of the BBB due to apoptosis in microvascular endothelial cells following exposure to high radiation doses [239]. While some insights into the molecular and cellular events underlying these defects have been gained, comprehensive data linking low IR exposure to an increased risk of AD remain limited. Therefore, further research is vital to elucidate the biological effects of IR at both high and low doses and to understand its potential role in the development of AD.

Finally, clinical data from long-term cancer survivors increasingly confirms that radiation-induced neuroinflammation acts as a “potent catalyst” for AD. Radiation does not just mimic AD; it accelerates the underlying molecular pathologies of the disease, particularly in older patients or those with genetic predispositions like the APOE4 allele through different mechanisms [66]:

1. The “Dual-Hit” Hypothesis: Amyloid and Tau

Radiation therapy creates a fertile environment for AD pathology through two primary inflammatory mechanisms:

‑ Seeding of Amyloid-β (Aβ): Radiation-induced vascular leakage impairs the glymphatic system and the BBB, reducing the clearance of Aβ. This leads to accelerated plaque deposition. In fact, radiation-induced damage to the Glymphatic System reduces Aβ clearance by 40% within six months of treatment [66].
‑ Tau Phosphorylation: The chronic oxidative stress following irradiation activates kinases (such as GSK-3β) that drive the hyperphosphorylation of tau proteins. “Radiation-Induced Tauopathy” has been identified as a distinct phenomenon where glia-driven inflammation spreads misfolded tau across distal brain regions [240].

2. Microglial Priming and AD Progression

The shift of microglia into a dysfunctional, pro-inflammatory state is a hallmark of both radiation injury and AD [67]. A recently published study identifies the NLRP3 inflammasome as a “seeding” mechanism where microglial activation post-radiation provides a physical scaffold for Aβ plaque aggregation [67]. A critical issue is the role of “primed” microglia:

‑ Loss of Homeostasis: Radiation forces microglia into a Disease-Associated Microglia (DAM) phenotype prematurely. These cells lose their ability to phagocytize (clear) Aβ plaques, instead releasing pro-inflammatory cytokines like IL-1β and TNF-α.
‑ Inflammasome Activation: Radiation triggers the NLRP3 inflammasome in microglia. This not only causes direct neuronal damage but also acts as a “scaffold” that promotes the aggregation of Aβ, effectively bridging the gap between radiation injury and Alzheimer’s progression.

3. Astrocyte Transformation and White Matter Loss

The transition of astrocytes to a neurotoxic (A1) phenotype drives the demyelination seen in late-stage radiation injury and AD [241]. Radiation-damaged microglia secrete IL-1α and TNF-α, which “flip” astrocytes into a neurotoxic state that destroys synapses and oligodendrocytes [241].

4. Vascular “Double-Jeopardy”

Because both AD and radiation injury target the neurovascular unit, patients experience a compounded effect [66]:

‑ Cerebral Amyloid Angiopathy (CAA): Radiation-damaged vessels are more susceptible to Aβ deposition. This weakens the vessel walls further, leading to microhemorrhages and localized ischemia, which are hallmark features of both advanced AD and late-stage radiation necrosis.
‑ Reduced Neurogenic Reserve: The inflammation in the hippocampal niche post-radiation destroys the progenitor cells required for memory formation, leaving the brain with zero “reserve” to combat the cognitive decline caused by AD-related atrophy.

5. Clinical Implications

‑ Pre-Symptomatic Screening: Patients undergoing cranial irradiation are now being screened for p-tau217 and other blood-based AD biomarkers to identify those at high risk for accelerated neurodegeneration.
‑ Targeted Anti-Inflammatories: Trials are investigating whether senolytic therapies (e.g., Dasatinib + Quercetin) can clear the senescent glia that drive both radiation damage and Alzheimer’s, potentially slowing the transition from cancer treatment to dementia [70].

### 3.3. Role of Ionizing Radiation in Amyotrophic Lateral Sclerosis

Amyotrophic lateral sclerosis (ALS) is a deadly neurodegenerative disorder associated with radiation exposure, particularly among veterans [242,243]. The majority of ALS cases are sporadic, with only 10–20% linked to a familial history. Recent research has concentrated on genetic mutations related to ALS, especially those affecting the antioxidant enzyme superoxide dismutase 1 (SOD1), highlighting the significance of oxidative stress in its pathogenesis [244].

The gene APEX1, associated with DNA repair, has also been implicated in ALS, providing neuroprotective effects against oxidative stress and IR exposure [245]. A study in a Scottish population identified amino acid mutations in the APEX1 gene among sporadic ALS patients [246]. The FUS gene has also been studied concerning ALS and radiation sensitivity, with knockouts revealing defective DNA repair mechanisms [247,248].

Epidemiological studies have reported increased ALS risk among individuals exposed to radiation, although some studies have found no significant associations [249]. Familial ALS cases often involve SOD1 mutations, which disrupt protein clearance and lead to DNA damage [250]. Although no point mutations in SOD1 have been linked to familial ALS, the entire SOD1 protein appears involved [251]. Protein misfolding may activate a cascade of events, including mitochondrial dysfunction and axonal transport alterations, ultimately leading to cell death [252].

Research by Poulletier de Gannes et al. [186] examined chronic exposure to electromagnetic radiation in an SOD1 mutant mouse model of ALS, finding no significant differences related to the development of ALS [187]. In similar studies, cells from patients with SOD1 mutations did not show differences in DNA double-strand break production after irradiation [188]. Further investigations are necessary to clarify the relationship between radiation exposure and ALS development.

### 3.4. Role of Ionizing Radiation in Parkinson’s Disease

Parkinson’s disease (PD) is the most prevalent neurodegenerative disorder, with its molecular mechanisms not yet fully understood. Aging, along with environmental and genetic factors, significantly contributes to PD etiology [253,254]. Key environmental risk factors include pesticides, herbicides, and metals, as well as IR [254]. IR can induce inflammation and stress responses in the nervous system, potentially leading to PD [255]. The CNS is particularly vulnerable to chemotherapy and IR [172]. Exposure to IR can result in cellular damage, affecting DNA repair and cell survival [256,257,258]. Moreover, IR exposure alters neurogenesis in neural precursor cells, which may contribute to cognitive impairment [144]. Oxidative stress, caused by increased ROS, damages neuronal cells, particularly dopaminergic neurons [66]. Mitochondrial dysfunction plays a crucial role in energy imbalances and cell death in PD progression [259]. Low doses of IR (under 5 Gy) can lead to mitochondrial dysfunction, affecting mitochondrial DNA and increasing oxidative stress [260,261]. Children and young adults are more susceptible to IR, highlighting the importance of analyzing radiation exposure’s effects across different age groups [261]. Mitochondrial damage due to IR is critical for neurodegeneration, as shown in various studies [262,263]. Additionally, IR exposure can lead to neuroprotective effects and activate reparative mechanisms in animal models [255]. Studies have demonstrated that neurotoxins like MPTP and pesticides can trigger Parkinson-like symptoms through mitochondrial dysfunction [264], and DNA damage has been implicated in PD as well. The radiosensitivity of PD patients’ cells indicates that genetic defects from somatic mutations during embryogenesis contribute to neuron death. IR can alter protein stability and promote the aggregation of a-synuclein, a key protein in PD [265]. This protein misfolding, alongside compromised mitochondrial function and ER stress, exacerbates PD pathology [266,267].

### 3.5. Preventive Strategies Related to Neurodegeneration

N-methyl-D-aspartate (NMDA) receptors are integral to various neurological processes, including synaptic development, maturation, plasticity, neural network activity, and overall cognitive functions [268]. Memantine Hydrochloride, which acts as an NMDA receptor antagonist, is utilized in the treatment of moderate to severe vascular dementia and AD [269]. Research involving animal models has demonstrated that the combination of memantine with hyperbaric oxygen therapy during radiotherapy can effectively repair white matter damage and promote neurogenesis. These interventions have been associated with improvements in anxiety-like behaviors and cognitive impairments observed in mice subjected to such treatments. Furthermore, clinical trials employing neuroimaging techniques and neuropsychological assessments have substantiated that memantine can enhance higher cognitive functions in patients undergoing cranial radiotherapy [270]. In a large-scale randomized, double-blind, placebo-controlled trial, memantine did not demonstrate a significant enhancement in memory retention compared to placebo among patients with brain metastases receiving WBRT. Nonetheless, memantine was linked to a delayed onset of cognitive decline and exhibited a favorable tolerability profile [213]. The hippocampus, a critical region for memory formation, contains cells in its dentate gyrus that are particularly susceptible to radiation damage, especially during radiotherapy for nasopharyngeal cancer [147]. The degree of damage sustained by the hippocampus serves as an indicator of the duration of cognitive dysfunction, emphasizing the importance of hippocampal neurogenesis in alleviating cognitive impairments associated with radiotherapy [142,271]. Therefore, implementing strategies aimed at protecting the hippocampus is essential for mitigating the cognitive impairments induced by radiation treatment.

A phase III multi-center clinical trial demonstrated that hippocampal-avoidance prophylactic cranial irradiation (HA-PCI) effectively reduced hippocampal atrophy in patients diagnosed with small-cell lung cancer, with positive results observed at both 4 and 12 months post-treatment. However, the trial did not establish a significant correlation between the extent of hippocampal atrophy and memory decline, as assessed by the Hopkins Verbal Learning Test-Revised (HVLT-R). This finding continues to fuel ongoing debates regarding the overall benefits of HA-PCI in clinical practice [272].

In a separate investigation, a notable reduction in the incidence of RN was observed when treating medium-sized brain metastases with hypofractionated SRS compared to the single-fraction SRS technique for lesions measuring 2.5–3 cm [273].

However, while Hippocampal Avoidance (HA) during WBR) is the gold standard for preserving memory in 2026, its implementation faces significant technical and biological hurdles. Recent clinical data suggest that the effectiveness of HA is often limited by “real-world” anatomical and oncological constraints, as in the following points [149,274]:

1. Technical and Dosimetric Challenges

Achieving a steep dose gradient between the target tissue and the hippocampus remains a primary obstacle [275]:

‑ Dose Fall-off Constraints: To effectively spare the subgranular zone, the dose must drop significantly over a few millimeters. In patients with tumors proximal to the temporal lobes, maintaining the therapeutic dose to the tumor while keeping the hippocampal dose below the 2026 safety threshold (typically D100% < 9 Gy) is often mathematically impossible.
‑ Contouring Variability: Even with AI-auto-segmentation tools, the hippocampus is a small, complex structure. Variability in manual or semi-automated contouring can lead to “geographic miss,” where the most sensitive neurogenic regions are inadvertently irradiated.

2. Patient-Specific Biological Factors

The “one-size-fits-all” approach to HA is being challenged by individual patient profiles [276]:

‑ The “Seed and Soil” Risk: There is a persistent clinical fear of peri-hippocampal recurrence. In 2025, approximately 5–8% of patients receiving HA-WBRT showed brain metastases within the 5 mm “avoidance zone,” raising concerns that sparing the “soil” (hippocampus) also spares the “seeds” (cancer cells).
‑ Baseline Cognitive Reserve: Patients with pre-existing vascular disease or early stage Alzheimer’s (confirmed via 2026 p-tau217 blood tests) may not benefit from HA. If the neurogenic niche is already compromised by age or systemic inflammation, the “sparing” of the hippocampus yields negligible cognitive gains [276].

3. Anatomical Limitations

‑ Brain Shift and Deformation: Throughout a 3-week course of radiation, the brain’s anatomy can change due to tumor shrinkage or steroid-induced changes in edema. Standard rigid masks may not account for these sub-millimeter shifts, potentially moving the hippocampus back into the high-dose zone [277].
‑ Surgical Cavities: If a patient has had a prior resection of a tumor near the hippocampus, the anatomical distortion makes precise sparing technically unreliable [278].

To overcome these challenges, clinical protocols are moving toward:

‑ Adaptive Radiotherapy (ART): Utilizing daily Online-CT or MR-Linac imaging to re-plan the dose distribution in real-time, accounting for daily anatomical shifts.
‑ Proton Therapy: Increasing use of Proton Beam Therapy to leverage the Bragg Peak, allowing for a near-zero “exit dose” through the hippocampus compared to traditional X-rays.

Recently, the focus of neuroprotection has shifted from broad anti-inflammatories to niche-specific preservation. Current research targets the biochemical “soil” of the hippocampus to ensure that neural stem cells (NSCs) not only survive radiation but remain capable of functional integration. Ongoing Clinical Trials are addressing several strategies:

1. Pharmacological Niche Stabilizers

Rather than blocking all inflammation, new agents aim to maintain the “pro-neurogenic” signaling balance.

‑ PPAR-δ Agonists (e.g., GW501516): Phase II trials in 2025 are evaluating these agents for their ability to suppress microglial activation specifically within the subgranular zone, thereby preventing the “gliogenic shift” where stem cells erroneously turn into astrocytes instead of neurons [279].
‑ Senolytics (D + Q Therapy): A major multi-center trial is testing the combination of Dasatinib and Quercetin to clear senescent endothelial cells and glia from the hippocampal niche immediately following radiotherapy. The goal is to prevent the “Senescence-Associated Secretory Phenotype” (SASP) from poisoning the local environment for new neurons [280].

2. Regenerative Medicine and “Niche Seeding”

For patients where damage has already occurred, research is exploring active restoration:

‑ Exosome-Based Therapies: Preclinical studies are using stem-cell-derived extracellular vesicles (EVs). These EVs are delivered intranasally to bypass the BBB, providing neurotrophic factors like BDNF and GDNF directly to the hippocampus to “jumpstart” neurogenesis [281].
‑ Human Neural Stem Cell (hNSC) Transplantation: While still in early-phase safety trials, researchers are investigating the transplantation of hNSCs into the hippocampal fimbria. In models, these cells have shown the ability to migrate into the irradiated dentate gyrus and restore spatial memory.

3. Neuromodulation and Behavioral Synergy [282]

Research is increasingly combining technology with biology to maximize neurogenic output:

‑ Non-Invasive Brain Stimulation (NIBS): Trials using Transcurrent Magnetic Stimulation (TMS) focused on the hippocampal-cortical network are being used alongside HA-WBRT. The goal is to provide “electrical enrichment” that encourages newly formed neurons to survive and wire into functional circuits.
‑ Digital Therapeutics: Integrated protocols now combine radiation with “Cognitive Priming” apps, which use specific spatial-navigation tasks to stimulate the Vascular-Neurogenic Niche during the window of highest plasticity.

The “next-gen” of neuroprotection is defined by multi-modal synergy:

‑ Physical Sparing (Hippocampal Avoidance);
‑ Chemical Shielding (PPAR-δ or Senolytics);
‑ Active Recovery (Exercise and BDNF-mimetics).

Some strategies addressed microglia, focusing on “re-tuning” rather than suppressing microglia [283,284,285]:

‑ Metabolic Modulators: Drugs like Metformin or Idebenone are being tested to force microglia back into an oxidative phosphorylation state, thereby restoring their homeostatic, neuroprotective functions.
‑ TREM2 Modulation: Since TREM2 is central to the transition into a protective phagocytic state, agonists are being investigated to enhance the clearance of radiation-induced cellular debris without triggering a cytokine storm.

Another target for treatment is vascular damage. In fact, current management is shifting from broad steroids to precision vascular stabilizers [78,81]:

‑ Anti-VEGF Therapy: Bevacizumab remains a primary treatment for radiation necrosis by reducing vessel fenestrations and permeability.
‑ STING Inhibitors: New nanodrugs (e.g., Pep-Cu5.4O@H151) target the cGAS-STING pathway to block the inflammatory cascade at the source of mtDNA leakage.
‑ Mitochondrial Protectants: Pre-treatment with agents like Idebenone has shown promise in 2025–2026 models for maintaining endothelial health and preventing barrier compromise.

Finally, microbiome modulation has been proposed a preventive strategy for radiation-induced cognitive disorders, through several mechanisms [38,286,287,288,289]:

1. Fecal Microbiota Transplantation (FMT): clinical and preclinical trials have shown that FMT can significantly restore gut permeability and improve cognitive performance following cranial radiotherapy.
2. Engineered Probiotics: New efforts involve genetically modifying strains like *E. coli* Nissle 1917 to produce barrier-enhancing metabolites like succinate, specifically to minimize radiation-induced syndrome and secondary neurodegeneration.
3. Dietary and Prebiotic Interventions: High-fiber diets and specific prebiotics are being studied for their ability to promote “radioprotective” species like Lachnospiraceae, which produce anti-inflammatory metabolites that mitigate the gut-liver-brain axis inflammatory response.
4. Depletion Strategies: Interestingly, some studies suggest that temporary depletion of gut flora (via antibiotics) immediately after irradiation may act as a “protective modulator,” suppressing the production of pro-inflammatory factors that drive brain injury.

## 4. IR as Treatment for Neurodegenerative Diseases

The role of IR in neurodegeneration is discussed and besides its role on triggering or worsening this process, there are also opposite evidence [290]. Recent evidence suggests that IR may play a role in the progression of neurodegenerative diseases, particularly those involving amyloidogenesis. The APP generates amyloid-beta (Aβ) peptides, which, due to an imbalance in production and clearance mechanisms, accumulate abnormally. This accumulation is associated with oxidative stress, NFTs, and neuronal loss, ultimately leading to cognitive impairment [227,229]. Although neurons generally resist radiation-induced cell death, studies have shown that low doses of IR can lead to cognitive dysfunction without significant changes in brain morphology. Conversely, higher doses produce observable microscopic changes [230].

The composition and location of toxic amyloid aggregates vary among neurodegenerative diseases. For instance, AD brains accumulate Aβ and tau aggregates, while α-synuclein aggregates are found in PD and huntingtin in Huntington’s disease (HD). These aggregation-prone proteins often contain sticky β-sheets or disordered domains, making them prone to misfolding and self-association [291,292].

The role of amyloids in neurodegeneration is complex and still debated. While large amyloid fibrils were initially thought to be toxic, recent perspectives suggest that soluble oligomers may be the most neurotoxic species [292,293,294]. Although targeting amyloid aggregation has shown promise in animal models, clinical trials have often failed to yield significant cognitive improvements [293,295,296,297].

IR has long been used in cancer treatment due to its ability to damage tumor cells while minimizing toxicity to healthy tissues. Recent research suggests that IR may also provide hormetic benefits, where low doses can induce protective biological responses. Low-dose irradiation has been shown to increase antioxidant levels and activate DNA repair mechanisms, enhancing resistance to subsequent DNA damage [185,298,299,300].

Studies indicate that low-dose radiation can modulate immune responses, promoting M2 polarization in macrophages, which leads to reduced inflammation [301,302,303,304,305,306,307,308,309,310,311]. These effects suggest that low-dose RT could have therapeutic implications beyond cancer treatment.

RT has a historical use in treating amyloidosis, a condition characterized by abnormal protein deposits that can lead to organ dysfunction [312,313,314]. Various forms of RT, including fractionated doses, have been employed to treat localized amyloidosis, demonstrating improvements in symptoms and reductions in amyloid deposits.

Despite its success in extra-cranial amyloidosis, RT has only recently been considered for neurodegenerative disorders. The mechanisms of RT’s potential benefits in neurodegeneration remain unclear, as high-dose RT typically kills cells and exacerbates oxidative stress. However, emerging evidence indicates that certain RT modalities, particularly low-dose RT, may provide protective effects against neurodegeneration [255].

In preclinical studies, low-dose radiation (0.1 Gy) did not induce AD-like pathologies or memory impairments in mice [315]. Moreover, low-dose RT has been shown to stimulate the proliferation and differentiation of neural stem cells in the hippocampus [316].

The mechanisms by which RT may alleviate amyloidosis are still being investigated. One hypothesis suggests that ionizing radiation can disrupt amyloid aggregates by breaking down intermolecular hydrogen bonds critical to their structure. Furthermore, RT may induce autophagy, enabling cells to eliminate toxic proteins and damaged organelles [317,318,319]. However, the dual nature of protein aggregation poses challenges in predicting whether RT will have beneficial or detrimental effects on neurodegenerative disorders.

In vitro studies have shown that low-dose radiation can improve cell viability in neurons exposed to Aβ, reducing neuroinflammatory cytokine production [320]. In animal models, irradiating AD mice with X-rays resulted in significant decreases in both the number and size of Aβ plaques, particularly with fractionated dose schemes [321].

In another study, low-dose irradiation improved cognitive function and reduced neuroinflammation in AD mice, while also decreasing levels of hyperphosphorylated tau [322]. Additionally, charged particle irradiation studies indicated that certain doses could effectively reduce amyloid aggregation and improve cognitive outcomes without causing significant damage to surrounding healthy tissues [323].

Initial indications of RT’s potential benefits for AD and PD emerged from case reports indicating improved cognitive and physical status in patients receiving multiple CT scans [324,325,326]. These findings led to pilot clinical trials exploring low-dose RT in AD patients, with some showing immediate improvements in cognitive function [327]. Current clinical trials are underway to evaluate the safety and efficacy of low-dose RT in mild to moderate AD patients, employing neurocognitive tests and imaging to assess outcomes [328,329,330].

Protein misfolding, amyloid aggregation, oxidative stress, and neuroinflammation are central hallmarks of neurodegenerative diseases such as AD and PD. The effectiveness of traditional therapies targeting these pathways has been mixed, highlighting the need for innovative approaches. Current evidence suggests that specific RT strategies may reduce or prevent these pathological features and improve clinical symptoms. While conventional high-dose RT may be too aggressive for neurodegenerative conditions, low-dose RT and new modalities like FLASH or proton therapy offer promising avenues for treatment. Further research is necessary to elucidate the underlying mechanisms of ionizing radiation’s effects in the context of neurodegeneration and to explore its therapeutic potential.

## 5. Conclusions

This review provides a comprehensive examination of the mechanisms and consequences of radiation-induced neurodegeneration in patients undergoing radiation therapy, particularly in the context of craniofacial tumors and BM.

Radiation therapy, while effective in treating tumors, can lead to significant neurodegenerative effects due to vascular injury, glial cell activation, and neurogenesis impairment. The review highlights the biphasic nature of radiation-induced vascular changes, which initially involves apoptotic processes followed by chronic alterations that compromise brain perfusion.

Chronic neuroinflammation driven by activated microglia and astrocytes contributes to cognitive deficits following radiation exposure. The review emphasizes the need to understand the inflammatory pathways involved, as prolonged microglial activation can exacerbate neuronal loss and cognitive decline.

Radiation significantly impairs neurogenesis in the hippocampus, an area critical for memory and learning. It disrupts the differentiation of neural precursor cells into mature neurons, which may lead to long-term cognitive impairments.

The effects of radiation on cognitive function are influenced by patient age and the specifics of the radiation treatment regimen. Younger patients show heightened sensitivity to neurocognitive deficits, necessitating careful treatment planning to minimize exposure to radiosensitive brain regions.

Genetic variations and specific biomarkers present in CSF can help predict individual susceptibility to radiation-induced neurotoxicity. Identifying these factors may enhance personalized therapeutic approaches and improve patient outcomes.

Emerging strategies, including neuroprotective agents, anti-inflammatory treatments, and advanced radiation techniques like proton therapy and hippocampal avoidance, show promise in mitigating cognitive decline associated with radiation therapy.

The potential role of ionizing radiation in exacerbating neurodegenerative diseases, such as AD and PD, is highlighted. The review suggests that understanding the interplay between radiation exposure and neurodegeneration is crucial for developing preventive strategies and therapies.

## Figures and Tables

**Table 1:** Model of RIBI cascade hubs and clinical consequences.

Central Hub	Molecular/Cellular Mechanism	Resulting Pathology
Vascular	Endothelial Senescence → BBB Leakage	Vasogenic Edema
Immune	NLRP3 Inflammasome → A1 Astrocytes	Synaptic Stripping
Metabolic	Hypoxia → HIF-1α → Aberrant VEGF	Radiation Necrosis
Cognitive	NSC Apoptosis → Gliogenic Shift	Memory Failure

**Table 2:** Main features of early and late stages of RIBI.

Stages	Cellular Activation
Early Stage: The Response to Acute Insult In the immediate aftermath of radiation, the glial response is primarily restitutive:	Microglia: Act as rapid-response sensors. They migrate to sites of endothelial cell death to clear cellular debris, preventing the spread of secondary “bystander” damage
	Astrocytes: Focus on ion and water homeostasis. Through the Glymphatic System, astrocytes attempt to flush out radiation-induced metabolic waste. Early astrocytic activation is often reversible and maintains the BBB integrity.
Late-Stage: The Transition to Chronic Degeneration. It is fueled by “Glial Senescence”:	A1 Astrocytic Transformation: Chronic inflammation causes astrocytes to lose their homeostatic functions (like glutamate uptake) and gain neurotoxic properties. These “A1” astrocytes actively contribute to the death of oligodendrocytes, driving the demyelination characteristic of late-stage injury.
	Microglial Exhaustion & Maladaptation: Late-stage microglia exhibit a Senescence-Associated Secretory Phenotype (SASP). They remain in a “primed” state, where even minor systemic infections (via the gut–brain axis) trigger exaggerated inflammatory responses, leading to the cognitive decline observed in clinical cohorts.
	The Glial Scar: Chronic astrogliosis results in physical and chemical barriers. While these scars initially “wall off” necrotic radiation zones, they eventually prevent the migration of neural stem cells, halting any possibility of endogenous repair.

**Table 3:** Temporal Comparison of Glial Roles.

Feature	Early Stage (Days to Weeks)	Late-Stage (Months to Years)
Primary Driver	Direct DNA damage & ROS	Chronic vascular leakage & SASP
Microglial Role	Debris Clearance: Phagocytosis of apoptotic neurons and endothelial cells.	Chronic Priming: Transition to a Disease-Associated Microglia (DAM) phenotype that releases neurotoxins.
Astrocytic Role	BBB Support: Upregulation of Aquaporin-4 (AQP4) to manage acute vasogenic edema.	Glial Scarring: Formation of dense “hemic scars” that block axonal regeneration and metabolic exchange.
Interaction	Synergistic repair signaling (e.g., IL-10).	Pathological loop; microglia induce A1 neurotoxic astrocytes via IL-1α and TNF-α.
Outcome	Neuroprotection/Homeostasis attempt.	Leukoencephalopathy and irreversible white matter necrosis.

**Table 4:** Late Effects of Ionizing Radiation.

Source/Species	Late Effect
Radiotherapy/human	Multiple lesions detected in the periventricular area, centrum semiovale, and corpus callosum via MRI; developed multiple sclerosis [181].
Radiotherapy/human	MRI showed new hyperintense lesions; exacerbation of multiple sclerosis triggered by radiation treatment [182].
X-radiation/human	Reactivation of quiescent MS with plaques confined to radiation fields; multiple sclerosis activated by X-radiation [183].
X-radiation (4000–6000 rad/40–60 Gy)/human	Poor clinical outcomes in patients receiving full tumoricidal doses, indicating high injury risk in patients with demyelinating disease [184].
Gamma-irradiation (0.5 Gy, weekly for 4 weeks)/mice	Suppression of pro-inflammatory cytokines, reduction of cytotoxic T cells, and induction of regulatory T cells observed [185].
50 Hz magnetic fields (100 and 1000 microT for 7 weeks)/mice	No association found between exposure and ALS development [186].
X-ray irradiation (0.8–1.5 Gy/min, total 4–16 Gy)/mice	No significant differences in DNA double-strand break production [187].
Dose-rate (1–2 Gy/min)/Cells from ALS patients	No significant differences in double-strand break production noted [188].
Continuous radiation (1.4 mGy/h for 45 days)/mice	Chronic low-dose radiation exposure found to be genotoxic [189].
Conventional radiotherapy/human	Direct correlation between radiation exposure and incidence of cerebrovascular events [190].
Gamma and X-rays (doses > 0.1 Gy)/human	Increased stroke risk associated with radiation exposure exceeding 0.1 Gy [191].
X-rays (0 to 30 Gy)/human	Increased adhesiveness of human aortic endothelial cells mediated by chemokines [192].
X-rays and gamma rays in interventional procedures/human	Increased stroke incidence observed among healthcare workers exposed to radiation [193].
Head and Neck Radiotherapy/human	Increased incidence of cerebrovascular events post-treatment [194].
Longitudinal studies of Japanese atomic bomb survivors	Increased incidence of cardiovascular diseases, including stroke and ischemic heart disease [195].
Single radiation dose of 14 Gy/ApoE−/− mouse	Irradiation accelerates development of inflammatory atherosclerotic lesions prone to hemorrhage [196].
Mean dose 97 mV followed by max of 909 mV gamma radiation/human	Increased risk of death due to cerebrovascular events compared to other cardiovascular instances [197].
CNS irradiation (0, 5, 15, 25, and 35 Gy)	Increased ICAM-1 expression suggests exacerbated inflammation due to leukocyte trafficking into the CNS [198].
Cath lab radiation exposure	Decreased telomerase length and increased thickness of carotid intima [199].

**Table 5:** Cognitive issues in animal models and humans.

Cognitive Domain	Animal Model Observation (2025–2026)	Clinical Human Equivalent
Spatial Navigation	Failure in the Morris Water Maze or Barnes Maze, where rodents cannot recall the location of escape platforms despite repeated training [223].	Patients report getting lost in familiar environments or difficulty navigating new hospital layouts.
Pattern Separation	Inability to distinguish between two similar but distinct contexts in Touchscreen Visual Discrimination tasks [66].	Difficulty multitasking or distinguishing between similar appointments/medication schedules.
Declarative Memory	Significant deficits in Novel Object Recognition (NOR); animals fail to spend more time with a new object, indicating a failure to form an “identity memory” [224].	Rapid forgetting of conversations, names, or events that occurred recently.

## Data Availability

No new data were created in this paper.
